# Dynamic evaluation of the contractile function of lumbodorsal muscles during locust pose in yoga by real-time ultrasound

**DOI:** 10.1186/s13102-021-00313-0

**Published:** 2021-08-10

**Authors:** Wenfen Liu, Jiachun Li, Xiang Zhou, Ningning Chen, Hui Ouyang, Zuofeng Xu, Yongsheng Zhu

**Affiliations:** 1 Shenzhen Hospital, Southern Medical University, Shenzhen, China; 2 The Third School of Clinical Medicine, Southern Medical University, Guangzhou, China; 3 Department of Ultrasound, The Seventh Affiliated Hospital of Sun Yat-Sen University, 628# Zhenyuan Road, 518100 Shenzhen, Guangdong China; 4 Department of Orthopedics, The Seventh Affiliated Hospital of Sun Yat-Sen University, 628# Zhenyuan Road, 518100 Shenzhen, Guangdong China; 5 Department of Microsurgery, Orthopedic Trauma and Hand Surgery, The First Affiliated Hospital, Sun Yat- sen University, No. 58, Zhongshan 2nd Road, 510080 Guangzhou, China; 6 Department of Gastroenterology, The Seventh Affiliated Hospital of Sun Yat-Sen University, 628# Zhenyuan Road, 518100 Shenzhen, Guangdong China; 7 Department of Ultrasound, Shenzhen Hospital, Southern Medical University, No. 1333, Xinhu Road, Xinan Street, Baoan District, Shenzhen, China

**Keywords:** Lumbodorsal muscles, muscle thickness, locust yoga pose, ultrasonography, low back pain

## Abstract

**Background and Purpose:**

Chronic low back pain (CLBP), which has a close relationship with lumbar muscle degeneration, can be effectively treated by exercise therapy, and yoga has been widely accepted by clinicians and patients with CLBP. The purpose of this study was to observe the changes in the thickness of lumbodorsal muscles that occur during locust pose in yoga and how these changes occur. From the changes in muscle thickness that occur in the locust pose, the contractile function of lumbodorsal muscles can be evaluated.

**Methods:**

Fifty-two healthy volunteers (from May 2019 to August 2019, age from 28 to 68 years, 23 males and 29 females (age: 40 ± 8 years; weight: 68.3 ± 5.2 kg; height: 170.2 ± 13.1 cm) were recruited, and lumbodorsal muscle, including the multifidus, longissimus, iliocostalis, and quadratus lumborum, ultrasonic examinations were carried out in the relaxed and contracted states. The changes in the thickness of the lumbodorsal muscles in the relaxed and contracted states were dynamically observed by real-time ultrasound when subjects were performing the locust yoga pose. Then, the thicknesses of the muscles during the two states were measured to calculate the ratio of contraction of each muscle and determine the statistical significance of the change in thickness of each muscle.

**Results:**

The mean thickness of the left multifidus in the relaxed state was 1.32 ± 0.27 cm (95 % CI: 1.24 ~ 1.39), that in the contracted state was 1.60 ± 0.30 cm (95 % CI: 1.52 ~ 1.69) (obviously different between the relaxed and contracted states, *P* < 0.001), and those in the corresponding right side were 1.37 ± 0.31 cm (95 % CI: 1.29 ~ 2.46) and 1.68 ± 0.38 cm (95 % CI: 1.58 ~ 1.79) (*P* < 0.001), respectively. The mean thickness of the left quadratus lumborum in the relaxed state was 1.38 ± 0.32 cm (95 % CI: 1.29 ~ 1.47), that in the contracted state was 1.62 ± 0.40 cm (95 % CI: 1.50 ~ 1.73) (*P* = 0.001), and those in the corresponding right side were 1.30 ± 0.32 cm (95 % CI: 1.21 ~ 1.39) and 1.55 ± 0.41 cm (95 % CI: 1.44 ~ 1.67) (*P* = 0.001), respectively. The mean thickness of the left longissimus in the relaxed was 2.33 ± 0.51 cm (95 % CI: 2.19 ~ 2.47), that in the contracted state was 3.20 ± 0.61 cm (95 % CI: 3.03 ~ 3.37) (*P* < 0.001), and those in the corresponding right side were 2.34 ± 0.49 cm (95 % CI 2.20 ~ 2.48) and 3.26 ± 0.68 cm (95 % CI 3.07 ~ 3.45) (*P* < 0.001), respectively. The mean thickness of the left iliocostalis in the relaxed state was 1.88 ± 0.41 cm (95 % CI: 1.76 ~ 1.99), that in the contracted state was 2.34 ± 0.49 cm (95 % CI: 2.00 ~ 2.47) (*P* < 0.001), and those in the corresponding right side were 1.98 ± 0.40 cm (95 % CI: 1.87 ~ 2.09) and 2.44 ± 0.56 cm (95 % CI: 2.29 ~ 2.60) (*P* < 0.001), respectively. The mean contracted state/resting state (C/R) of the longissimus was 1.39 ± 0.14 on the left and 1.40 ± 0.16 on the right. The multifidus and iliocostalis had the second highest C/R. The mean C/R of the multifidus was 1.23 ± 0.12 on the left and 1.24 ± 0.15 on the right, and the mean C/R of the iliocostalis was 1.25 ± 0.12 on the left and 1.24 ± 0.14 on the right. The quadratus lumborum had the lowest C/R, and the mean C/R of the quadratus lumborum was 1.17 ± 0.10 on the left and 1.19 ± 0.11 on the right.

**Conclusions:**

Ultrasound can be used to dynamically assess the contractile function of the lumbar muscle in the locust pose of yoga, the C/R ratio can be used to indicate the ability of a muscle to contract, and dynamic ultrasound can guide lumbar exercise and feedback the exercise results. The establishment of this model allowed data regarding the contraction state of the lumbar muscle to be obtained in a normal population, and based on this, future studies can further explore and evaluate the contraction state of the lumbar muscle after yoga exercise in CLBP patients, the effect exercise on lumbar instability and on a patient population after lumbar operation.

## Introduction

Low back pain (LBP) is defined as pain and discomfort localized above the inferior gluteal folds and below the costal margin, regardless of whether leg pain is involved[1, 2]. Chronic LBP (CLBP) is defined as a minimum of 12 weeks of LBP [2], and it is the leading cause of years lived with disability in both developed and developing countries and sixth in terms of overall disease burden[3–5]. Despite the very high prevalence and severe consequences of this disease, its pathophysiology is poorly understood[6], systematic literature reviews have indicated that physical exercises are effective in reducing pain and disability in patients [7–9].

Exercise training is an effective treatment for nonspecific CLBP, the European guidelines released in 2006 recommend supervised exercise therapy as a first-line treatment in the management of this pain[10], however, the best mode of exercise training is unknown[2]. Yoga, which originated in ancient India and has been widely accepted by those clinicians and patients[11–18], could reduce pain better than nonexercise training comparators[19]. A practice guideline from the American College of Physicians and American Pain Society lists a specific style of yoga as having fair evidence of a moderate benefit [9, 11]. This is particularly useful when the new therapy may have other potential benefits, such as a lower cost[16]. However, the mechanism by which yoga relieves pain and dysfunction cannot be explained by the existing research.

CLBP is closely related to lumbar muscle degeneration [20–22]. Macroscopically, this muscle degeneration is characterized by a decrease in cross-sectional area [23–27] and an increase in the fat content [28–30] of the lumbar paraspinal muscles. In addition to macroscopic changes, it has been proposed that microscopic changes can also occur in patients with nonspecific LBP. Patients with severe pain have a higher portion of type IIX (fast twitch glycolytic, previously called type IIB) at the expense of type I (slow twitch oxidative) fibers [31]. Hence, the changes in fiber type, could lead to lowered fatigue resistance of the paraspinal muscles, which in turn results in higher vulnerability of the lumbar spine [32].

The lumbodorsal muscles are mainly composed of the multifidus, longissimus, iliocostalis and quadratus lumborum. Magnetic resonance imaging (MRI) is considered the standard method of imaging for the assessment of posterior trunk muscles. MRI can provide clear images of not only anatomical structures but also boundaries of different tissues. However, the long waiting time for an appointment, long examination time and high examination cost limit its use in routine clinical applications[33]. Additionally, it is difficult to dynamically evaluate the lumbar and back muscles by MRI.

Ultrasonography (US) has advanced rapidly over the past 50 years, and it is widely used in the clinic in fields such as gynecology and obstetrics and other fields in which abdominal and superficial organs, cardiovascular structures, and especially musculoskeletal structures are examined. For instance, US has been used to directly assess atrophy and hypertrophy in different muscles[34]. US imaging has obvious advantages over other imaging techniques: it is noninvasive, does not use radiation, can be used to acquire images in multiple planes, has low examination costs, and most importantly, can dynamically assess the shrinkage of lumbodorsal muscles during contractions and relaxation in real time, which cannot be achieved by MRI[33, 35]. Muscle thickness often increases by varying degrees as it contracts. Existing studies have reported that ultrasonography can be used to observe changes in the thickness of muscles, except for lumbodorsal muscles, during contractions and relaxation to detect or measure muscle “activity”[34, 36].

However, currently, there is no uniform, simple, feasible method for assessing lumbodorsal muscle activity. Does yoga yield significant changes in the lumbar muscle? Which muscle groups are most affected by a particular type of yoga? Are certain imaging methods appropriate for specific evaluations?

**Fig. 1:**
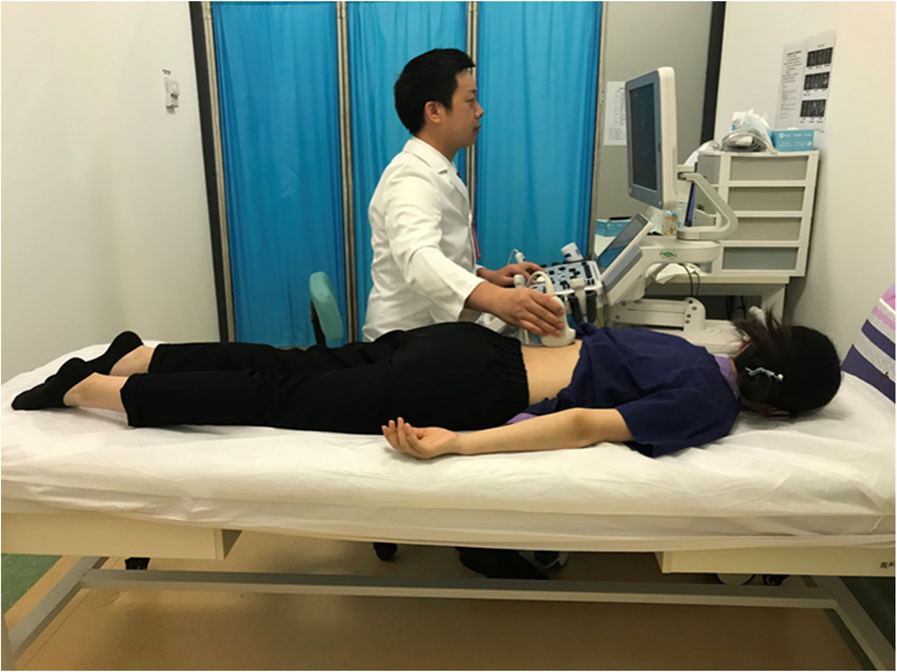
The head is lifted upward as much as possible, and both the upper limbs and lower limbs are stretched out from the bed surface. The lower abdomen is in contact with the bed surface to act as the fulcrum. The angle of the lower limbs and fulcrum and the angle of the upper body and fulcrum are close to 30°. The whole body is bent like an inverted arch.

Thus, we need to identify a way to objectively assess exercise effectiveness. We chose the locust yoga pose (Fig. 1) as the contracted state and the prone position as the resting state. The main purposes of this study were to use real-time ultrasound to observe the changes in the thicknesses of lumbodorsal muscles dynamically in contracted and relaxed states, to determine which muscle has the highest contraction ratio of change, and to determine whether ultrasound can be effective for examining the lumbar muscle.

This study provides a theoretical basis for further ultrasonic assessment of the changes in lumbodorsal muscles in patients with pain and of the changes in lumbodorsal muscles after yoga exercise. To provide imaging support for the diagnosis and differential diagnosis of CLBP, to evaluate of yoga exercise results, and the etiological mechanism can be further explored.

## Methods

From May 2019 to August 2019, a total of 200 volunteers (with no history of neurological, cognitive, metabolic, cardiovascular, pulmonary, or low back musculoskeletal impairment) volunteered to participate in this study and were recruited continuously and randomly from the Physical Examination Center of the Seventh Affiliated Hospital of Sun Yat-sen University. Fifty-two healthy volunteers, ranging in age from 19 to 68 (age: 40 ± 8 years; weight: 63.3 ± 5.2 kg; height: 170.2 ± 13.1 cm)) and including 23 males and 29 females, were included in the study. The other volunteers were excluded because they had current or chronic LBP; had a history of back surgery; had a serious spinal pathology, such as fracture, cancer, or infection; or did not agree to sign in the informed consent form. All participants underwent ultrasound to assess lumbodorsal muscles using a 3.6 MHz convex array probe with an ultrasound coupler (DC-6, made in 2018, Mindray, Mindray Biomedical Electronics Co., Ltd., Shenzhen, China).

Each eligible participant was informed of all experimental procedures and provided informed written consent. In addition, information about their height and weight was obtained before the examination. All subjects were trained to master the completely relaxed state and the posture of the locust yoga pose.

### Relaxed state

For the relaxed state, subjects laid flat on the examination bed in the prone position, with both of their arms resting naturally to the sides of their body, their head slightly resting to one side, and their legs relaxed.

### Locust pose

For the locust pose, participants laid in a prone position, with their hands pronated, shoulder blades retracted together, gaze looking forward and downward, shoulder and elbows extended, spine extended, lower abdomen in contact with the bed surface to act as a fulcrum, hips and legs extended, and feet plantarflexed; both the angle between the lower limbs and the bed surface and the angle between the trunk and the bed surface were maintained to be as close to 30° as possible. The whole body was bent like an inverted arch (Fig. 1).

### Ultrasound procedure

First, gel was applied for ultrasound, and in the relaxed state, the probe was placed transversely at the upper waist, and the 1st lumbar vertebra was identified according to the 12th rib. Then, the probe was gradually moved downward to observe the general shape of the cross-sections of each lumbodorsal muscle. At the level of the 4th lumbar vertebra, the probe was rotated 90° clockwise to view a section of each muscle in the sagittal plane. The transducer was oriented in the sagittal plane so that it was parallel to the muscle fibers to the greatest extent possible[37]. The muscles including the multifidus, longissimus, iliocostalis, and quadratus lumborum, were measured in the sagittal plane. Each muscle was measured as vertically as possible along the long axis of the muscle fibers to reduce measurement errors due to angulation. All eight muscles on both sides of the lower back were measured sequentially, from shallow to deep, from middle to side, first left and then right. The order was as follows: the multifidus, the quadratus lumborum, the longissimus and the iliocostalis.

### Measure method

Each subject was in a relaxed state when the optimal sagittal section of each muscle was recorded to ensure that the probe position did not change, and then, the subject was asked to perform the locust yoga pose. To record the optimal sagittal section of each muscle during the contracted state, still images were unified with the double mode[38], and still images in the left diastolic and right contracted states were assessed. The thickness of all the muscles in different positions was measured three times, and then, on the other contraction image, in the same position, the thickness was also measured three times. Finally, the average of the measurements was recorded.

### Statistical method

All examinations and measurements were performed by two sonographers with more than 10 years of ultrasound experience. All data analyses were performed using SPSS statistical software (v.20.0; IBM Corp., Armonk, NY). All data followed a normal distribution. One-way ANOVA was used to compare the differences between the two groups of data (pre-exercise and exercise motion), and the relationship between each of them was also analyzed. A p value of less than 0.05 was considered significant.

## Results

In this study, 52 individuals met the inclusion criteria, including 31 women and 21 men aged 18 to 68 years; the mean age was 40.35 ± 11.042 years, and the mean body mass index (BMI) was 23.845 ± 3.476. Clear ultrasound images were captured for all volunteers.

The thickness of the muscles, including the multifidus, longissimus, iliocostalis, and quadratus lumborum, in relaxed and contracted states was measured. The mean thicknesses of these muscles in the relaxed and contracted states are shown in Table 1. The mean thickness of the left multifidus in the relaxed state was 1.32 ± 0.27 cm (95 % CI: 1.24 ~ 1.39), and that in the contracted state was 1.60 ± 0.30 cm (95 % CI: 1.52 ~ 1.69) (obviously different between the relaxed and contracted states, *P* < 0.001). The mean thickness of the right multifidus in the relaxed state was 1.37 ± 0.31 cm (95 % CI: 1.29 ~ 2.46), and that in the contracted state was 1.68 ± 0.38 cm (95 % CI: 1.58 ~ 1.79) (*P* < 0.005). The mean thickness of the left quadratus lumborum in the relaxed quadratus was 1.38 ± 0.32 cm (95 % CI: 1.29 ~ 1.47), and that in the contracted quadratus lumborum was 1.62 ± 0.40 cm (95 % CI: 1.50 ~ 1.73) (*P* = 0.005). The mean thickness of the right quadratus lumborum in the relaxed quadratus was 1.30 ± 0.32 cm (95 % CI: 1.21 ~ 1.39), and that in the contracted quadratus lumborum was 1.55 ± 0.41 cm (95 % CI: 1.44 ~ 1.67) (*P* = 0.005). The mean thickness of the left longissimus in the relaxed state was 2.33 ± 0.51 cm (95 % CI: 2.19 ~ 2.47), and that in the contracted state was 3.20 ± 0.61 cm (95 % CI: 3.03 ~ 3.37) (*P* < 0.005). The mean thickness of the right longissimus in the relaxed state was 2.34 ± 0.49 cm (95 % CI: 2.20 ~ 2.48), and that in the contracted state was 3.26 ± 0.68 cm (95 % CI: 3.07 ~ 3.45) (*P* < 0.005). The mean thickness of the left iliocostalis in the relaxed state was 1.88 ± 0.41 cm (95 % CI: 1.76 ~ 1.99), and that in the contracted state was 2.34 ± 0.49 cm (95 % CI: 2.00 ~ 2.47) (*P* < 0.005). The mean thickness of the right iliocostalis in the relaxed state was 1.98 ± 0.40 cm (95 % CI: 1.87 ~ 2.09), and that in the contracted state was 2.44 ± 0.56 cm (95 % CI: 2.29 ~ 2.60) (*P* < 0.005).

**Table 1:** The mean thicknesses of four muscles in the relaxed and contracted states (Unit: centimeter), 95 % confidence interval (CI) in Resting state and Contracted state, the related C/R ratio, and the p value in differences between the relaxed and contracted states for the four muscles

	the left multifidus	the right multifidus	the left quadratus lumborum	the right quadratus lumborum	the left longissimus	the right longissimus	the left iliocostalis	the right iliocostalis
Resting state	1.32 ± 0.27	1.37 ± 0.31	1.38 ± 0.32	1.30 ± 0.32	2.33 ± 0.51	2.34 ± 0.49	1.88 ± 0.41	1.98 ± 0.40
Contracted state	1.60 ± 0.30	1.68 ± 0.38	1.62 ± 0.40	1.55 ± 0.41	3.20 ± 0.61	3.26 ± 0.68	2.34 ± 0.49	2.44 ± 0.56
95 % CI in Resting state	1.24 ~ 1.39	1.29 ~ 2.46	1.29 ~ 1.47	1.21 ~ 1.39	2.19 ~ 2.47	2.20 ~ 2.48	1.76 ~ 1.99	1.87 ~ 2.09
95 % CI in Contracted state	1.52 ~ 1.69	1.58 ~ 1.79	1.50 ~ 1.73	1.44 ~ 1.67	3.03 ~ 3.37	3.07 ~ 3.45	2.00 ~ 2.47	2.29 ~ 2.60
C/R	1.23 ± 0.12	1.24 ± 0.15	1.17 ± 0.10	1.19 ± 0.11	1.39 ± 0.14	1.40 ± 0.16	1.25 ± 0.12	1.24 ± 0.14
F	26.49	21.14	10.71	11.54	62.17	62.11	26.34	23.63
P	< 0.001	< 0.001	= 0.001	= 0.001	< 0.001	< 0.001	< 0.001	< 0.001

**Fig. 2:**
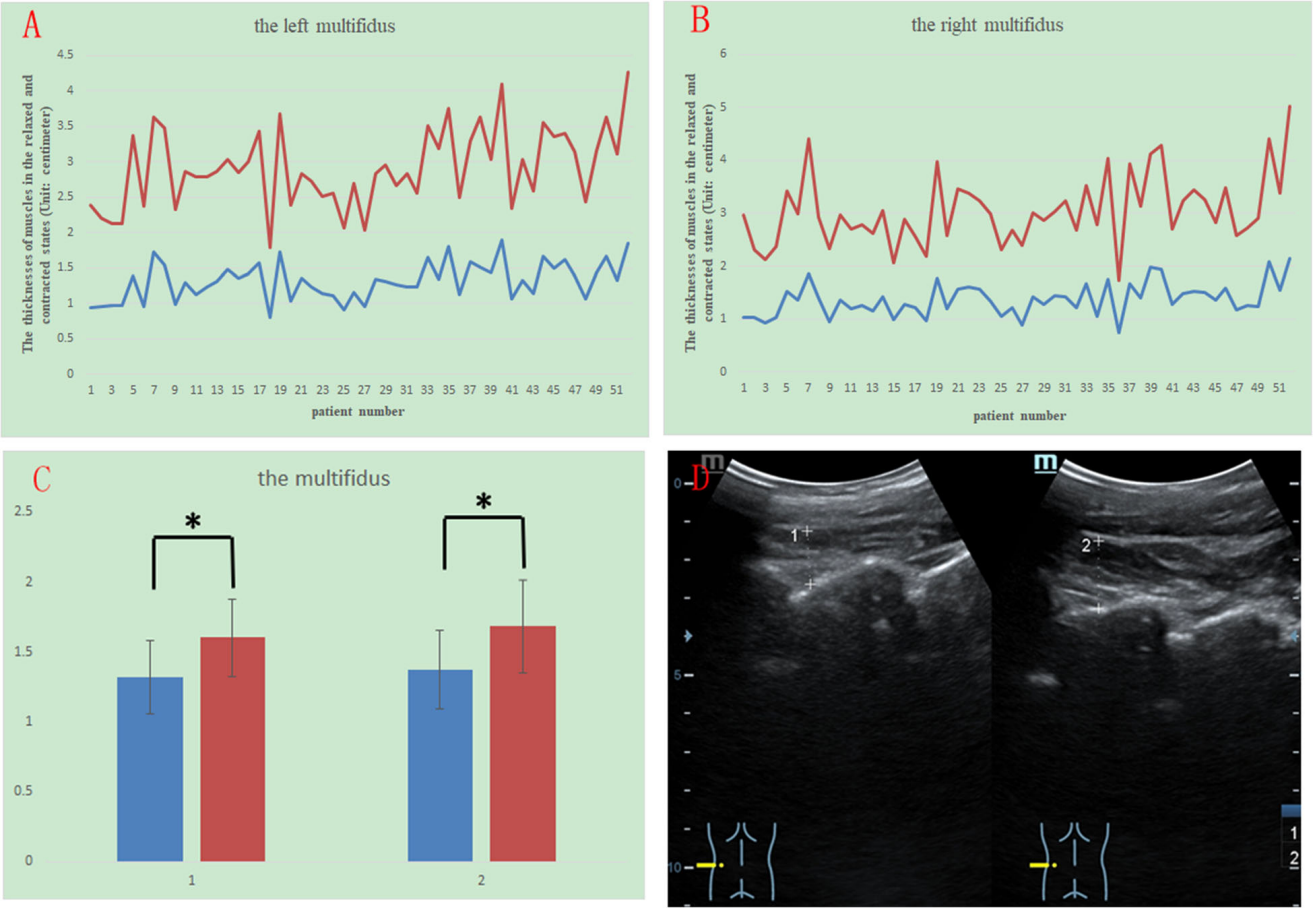
The thicknesses of the left (Fig. 2 A) and right (Fig. 2B) multifidus in the resting (blue line) and contracted conditions (red line). Comparison of the thicknesses in the resting (blue column) and contracted conditions (red column). Group 1 is right, and group 2 is left (Fig. 2 C). The sonogram of the multifidus: left shows the relaxed state, and right shows the contracted state (Fig. 2D)

**Fig. 3:**
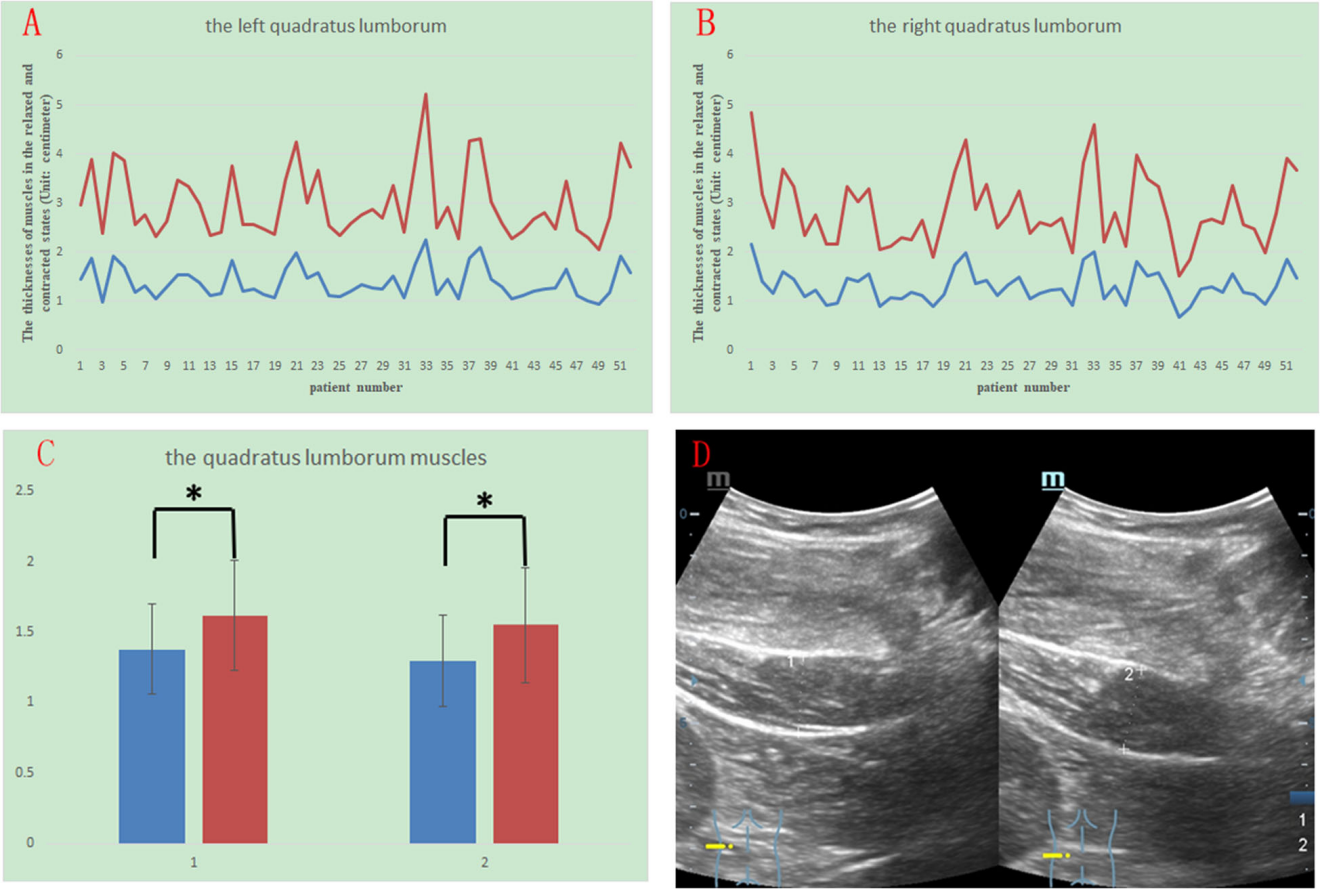
The thicknesses of the left (Fig. 3 A) and right (Fig. 3B) quadratus lumborum muscles in the resting (blue line) and contracted conditions (red line). Comparison of the thicknesses in the resting (blue column) and contracted conditions (red column). Group 1 is right, and group 2 is left (Fig. 3 C). The sonogram of the quadratus lumborum: left shows the relaxed state, and right shows the contracted state (Fig. 3D)

**Fig. 4:**
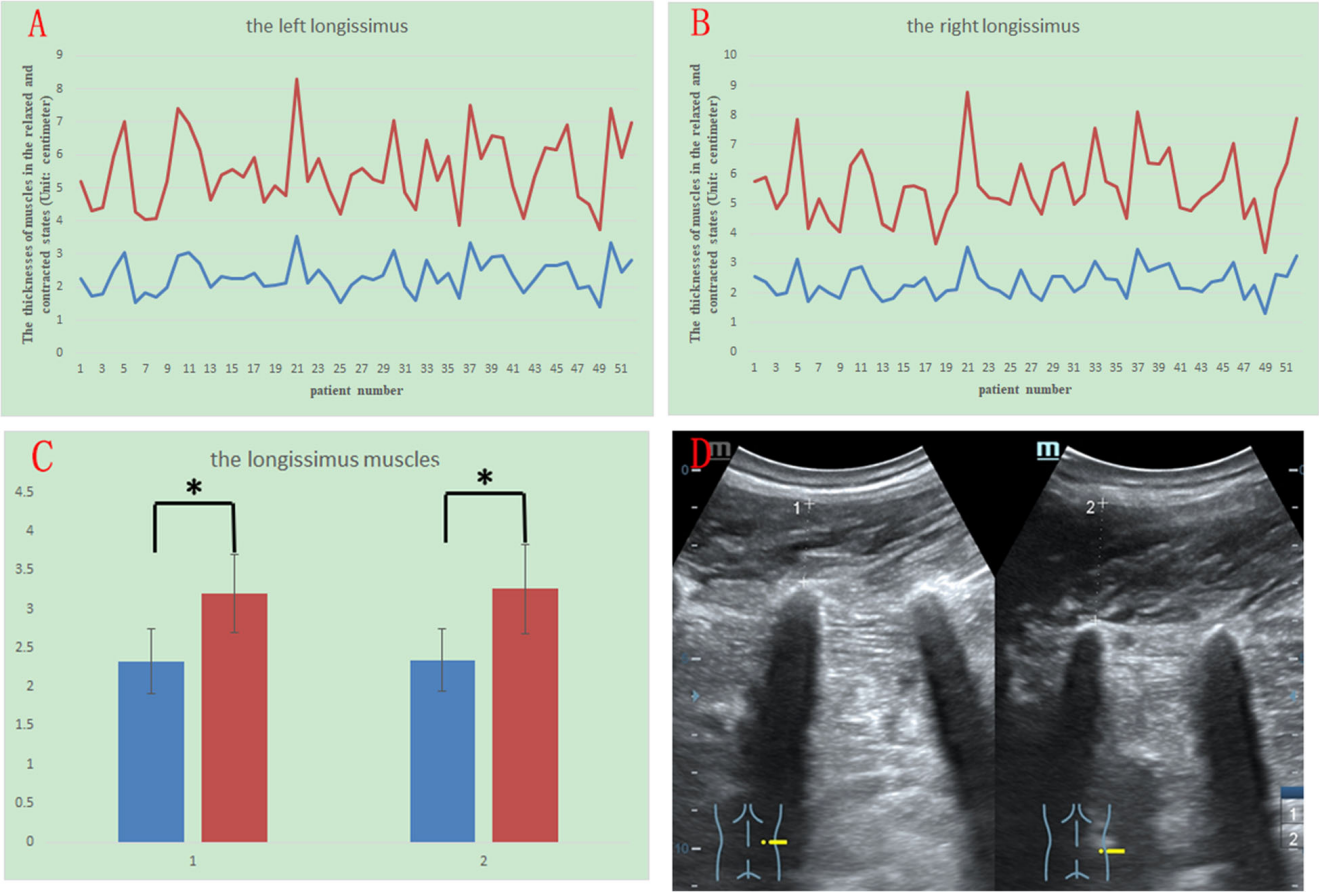
The thicknesses of the left (Fig. 4 A) and right (Fig. 4B) longissimus muscles in the resting (blue line) and contracted conditions (red line). Comparison of the thicknesses in the resting (blue column) and contracted conditions (red column). Group 1 is right, and group 2 is left (Fig. 4 C). The sonogram of the longissimus: left shows the relaxed state, and right shows the contracted state (Fig. 4D)

**Fig. 5:**
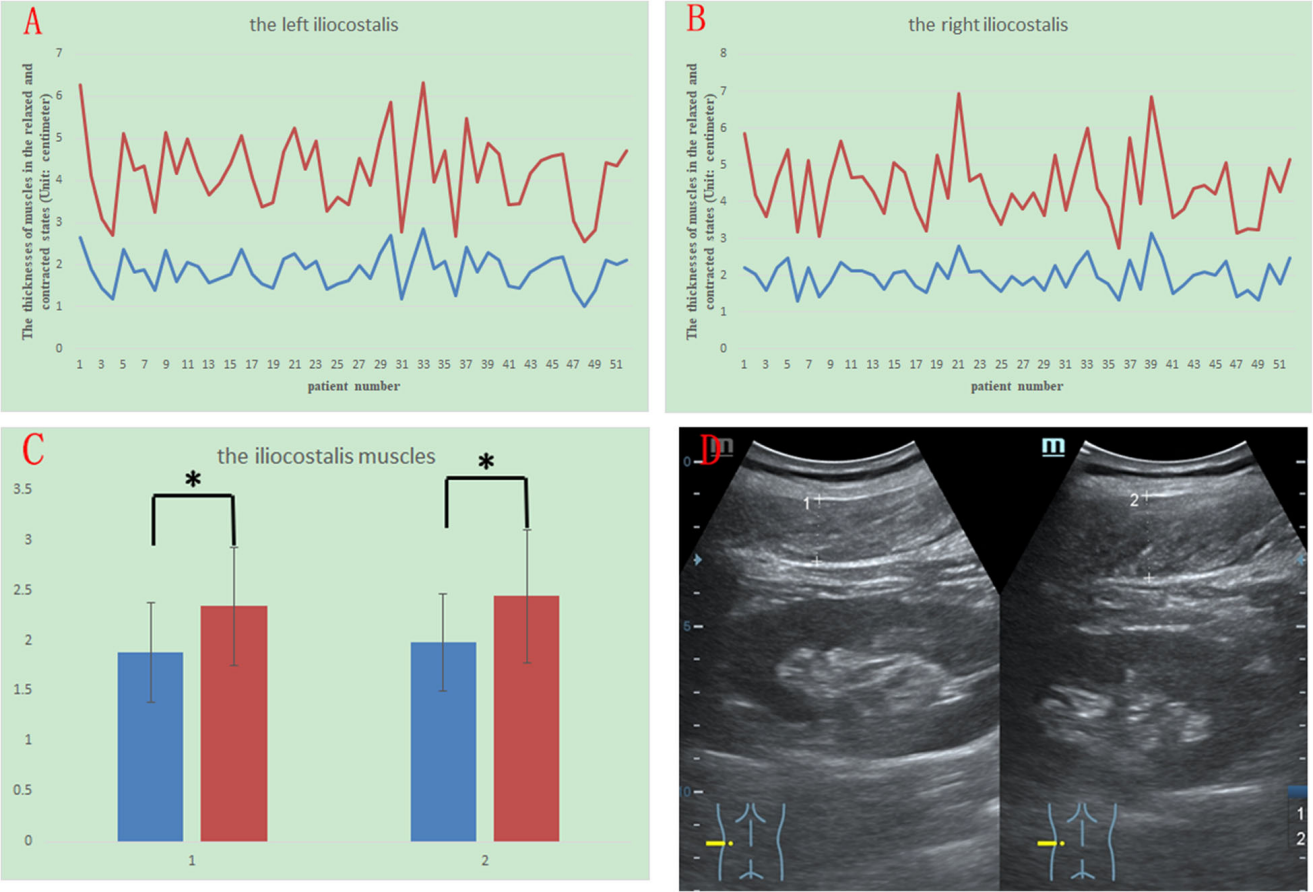
The thicknesses of the left (Fig. 5 A) and right (Fig. 5B) iliocostalis muscles in the resting (blue line) and contracted conditions (red line). Comparison of the thicknesses in the resting (blue column) and contracted conditions (red column). Group 1 is right, and group 2 is left (Fig. 5 C). The sonogram of the iliocostalis: left shows the relaxed state, and right shows the contracted state (Fig. 5D)

The thickness in the contracted state divided by that in the relaxed state is denoted by C/R, and the mean C/R for each muscle is also shown in Table 1. Among these four muscles, the longissimus had the highest C/R, and the mean C/R was 1.39 ± 0.14 on the left and 1.40 ± 0.16 on the right. The multifidus and iliocostalis had the second highest C/R. The mean C/R of the multifidus was 1.23 ± 0.12 on the left and 1.24 ± 0.15 on the right, and the mean C/R of the iliocostalis was 1.25 ± 0.12 on the left and 1.24 ± 0.14 on the right. The quadratus lumborum had the lowest C/R, and the mean C/R of the quadratus lumborum was 1.17 ± 0.10 on the left and 1.19 ± 0.11 on the right. The differences between the relaxed and contracted states for all the muscles had a p value of less than 0.005 (Fig. 2). Figures 2, 3, 4 and 5 show different muscles in the relaxed and contracted states. The blue line indicates the thickness of the muscle in the relaxed state. The red line indicates the thickness of the muscle in the contracted state.

## Discussion

In the past, researchers studying CLBP have comprehensively assessed the vertebrae, discs, and intervertebral joints of the back. Nevertheless, the importance of the lumbodorsal muscles in stabilizing the lumbar spine should not be underestimated[20, 39], and this viewpoint was excellently illustrated in a study that provided quantitative data about the stabilizing effects of muscles with regard to the mechanics of the spine. Moreover, previous studies have shown that the lumbar muscles often degenerate to different degrees when patients suffer from the pain[20]. Changes in structure may lead to changes in muscle fiber elasticity, which inevitably affect muscle function. Even without changes in muscle structure, pain causes pain-related nerve suppression, and the level of activity of lumbar muscles decreases to prevent tissue damage[40], thereby affecting muscle contractile function. Therefore, it can be assumed that patients with CLBP exhibit changes in muscle contractile function to a certain extent, regardless of whether there are morphological structural changes or how the structure changes.

Yoga has been widely accepted by clinicians, and patients could reduce pain with CLBP[11–18]. Kliziene et al. found that after eight months of core stability training, the cross-sectional area of the multifidus muscle increased by 22 %[41]. However, it is not clear how lumbar muscle contractions change when doing yoga, and the effects of locust yoga pose and its relation between muscle thicknesses are not clear.

As actin and myosin filaments overlap more during muscle contractions than during relaxation, the muscle generally becomes thicker and shorter[42, 43]. Real-time ultrasound can be used to observe dynamic changes in muscle thickness from relaxed to contracted states. The contraction ratio can be calculated according to the muscle thickness in the two different states to quantitatively evaluate muscle contractile function[34, 44].

Therefore, in this study, we focused on the changes in the lumbar muscles when people perform yoga. We used ultrasound to assess changes in lumbar muscle relaxation and contraction during the locust pose in a normal population. The establishment of this model allowed data regarding the contraction state of the lumbar muscle to be obtained in a normal population, and based on this, future studies can further explore and evaluate the contraction state of CLBP patients, the lumbar muscle after yoga exercise, the effect exercise on lumbar instability and on a patient population after lumbar operation.

In the existing literature on lumbodorsal muscles, the posture in which patients were examined differed among studies. Some evaluations were performed in an upright position, while others were performed in a prone or supine position. In the supine position, which is often used for MRI and CT scans, the back muscles are often compressed and deformed due to the patient’s body weight. In the upright position, the human body needs small levels of muscular activity to maintain the pose, which might affect the thicknesses of lumbar muscles[22]. Furthermore, in the standing and supine positions, it is difficult to observe and measure changes in lumbodorsal muscles with US. Therefore, the prone position was chosen for our study. With subjects in the prone position, we can ensure that there are no additional contractions of the muscles in the relaxed state, and it is convenient for taking measurements.

Yoga has been widely accepted, however, yoga contains a variety of postures[45], and we assume that each posture targets different muscles. According to our experience in clinical practice, the locust yoga pose is an effective exercise to relieve CLBP. We chose the locust yoga pose as the contracted state for the following three primary reasons. First, the locust yoga pose is effective in relieving pain. Second, the action is simple and easy to perform. Third, it is convenient for us to observe and measure muscles during this pose.

In our study, the data objectively showed that the locust yoga pose can be used to exercise the lumbar back muscles, especially the longissimus. According to the measurements of the thickness of lumbodorsal muscles taken in a uniform manner during the locust pose, the contraction ratio of each muscle (C/R) was calculated to uniformly quantify the contraction function of each muscle.

However, our study has some limitations. First, yoga involves various poses; in this study, we investigated only the locust pose. Second, the sample size was relatively small, so a study with a larger sample size needs to be conducted to determine the normal range of the contraction ratio of each muscle. Third, the subjects of this study were normal people without pain. Some patients may not be able to perform the exercise mentioned above and therefore may not be eligible for future studies. Additionally, this posture may not be suitable for US measurement of the lumbosacral muscles for patients, such as spinal stenosis, facet problems, tumors and fractures.

## Conclusions

Ultrasound can be used to dynamically assess the contractile function of the lumbar muscle in the locust pose of yoga, the C/R ratio can be used to indicate the ability of a muscle to contract, and dynamic ultrasound can guide lumbar exercise and feedback the exercise results. The establishment of this model allowed data regarding the contraction state of the lumbar muscle to be obtained in a normal population, and based on this, future studies can further explore and evaluate the contraction state of CLBP patients, the lumbar muscle after yoga exercise, the effect exercise on lumbar instability and on a patient population after lumbar operation.

## Data Availability

The datasets used or analyzed during the current study are available from the corresponding author on reasonable request.

## Abbreviations

BMI: body mass index
C/R: contracted state/resting state
CSAs: cross-sectional areas
LBP: low back pain
CLBP: chronic LBP
MRI: magnetic resonance imaging
US: ultrasonography
